# Editorial: Chronic Inflammatory Response Accompanying Neuronal and Axonal Degeneration Post Brain Injury

**DOI:** 10.3389/fncel.2022.930697

**Published:** 2022-06-22

**Authors:** Xintong Ge

**Affiliations:** ^1^Department of Geriatrics, Tianjin Medical University General Hospital, Tianjin, China; ^2^Tianjin Geriatrics Institute, Tianjin, China

**Keywords:** chronic inflammatory response, neurodegeneration, traumatic brain injury, stroke, Alzheimer's disease

Brain injury from trauma, stroke, and other neurological diseases could lead to chronic loss of neurons and axons in the brain, which results in neurological deficits. Recent studies showed that several biological processes, such as chronic inflammatory response, immune regulation, and deposition of pathological proteins play important roles in the occurrence and development of neuronal and axonal degeneration (Liang et al., 2017; Kempuraj et al., 2020; Kim et al., 2021). For example, chronic neuro-inflammatory response has been detected in animal models of traumatic brain injury, stroke, and Alzheimer's disease (AD). It is an extraordinarily significant factor that impacts neuropathology, especially progressive neurodegeneration (Heneka et al., 2015; Simon et al., 2017; Shi et al., 2019). However, the in-depth cellular and molecular mechanism underlying this pathological procedure has not been fully elucidated. This Research Topic aims to shed more light on the regulation mechanisms of chronic inflammatory response accompanying neuronal and axonal degeneration. Herein, four review articles provide new insights in the pathogenesis of chronic inflammatory response induced by brain injury, and the potential therapeutic strategies for impaired neurological function.

The first review on traumatic brain injury (TBI) (Shi et al.) highlighted the potential value of the Sigma-1 receptor (Sig-1R) as a therapeutic target for clinical translation. Sig-1R is a ligand-operated chaperone protein that is primarily located at the endoplasmic reticulum membrane. It can act as a pluripotent modulator in diverse pathological conditions, due to its versatile ligand affinity, unique cellular location, and multi-site translocation profile. Based on a large number of preclinical studies on the neuroprotective effects of Sig-1R, the authors reviewed the potential roles of Sig-1R in regulating neuroinflammation, oxidative stress, cellular calcium homeostasis, neural excitoxicity, endoplasmic reticulum stress, and mitochondrial dysfunction in secondary brain injury. From this, Sig-1R was believed to be a potential therapeutic target for TBI, which may expand the current narrow therapeutic window.

In the second review article (Yu et al.), the authors focused on the crosstalk between microglia activation and endogenous neuroplasticity, especially on the plastic alterations in the whole brain network, and their implications for structural and functional restoration after stroke. Recent advances in switching microglia phenotype polarization were summarized, in order to discuss the potential efficacy of microglia-based extrinsic restorative interventions in promoting recovery. Besides, in view of the widespread activation of microglia, and their various roles in regulating neuropathology after stroke, the authors suggested that a thorough understanding of the development of microglial activation in the post-stroke brain is urgently required. Several questions need to be addressed: (1) Why does microglial activation exhibit different spatiotemporal manners in distinct animal models and human brains? (2) How to switch microglia polarization to a favorable activation profile and coordinate them in the chronic stroke phase? (3) How to distinguish microglia and macrophages, especially when peripheral monocytes/macrophages infiltrate into the injured brain, and exhibit various phenotypes as well as distinct cytokine production? (4) How pathological conditions and baseline immunity in patients with rmTBI affect microglial activation?

The authors of the third review article focused on the relationship between blood-brain barrier (BBB) damage and amyloid pathology in AD (Wang et al.). Long-lasted BBB leakage has been widely reported in AD animal models. It was believed to be associated with chronic neuroinflammation in the whole brain and poor neurological outcomes. In this paper, the authors mainly reviewed the interaction between Aβ and BBB in AD from two expects: (1) How does Aβ destroy the integrity and function of BBB? (2) How does chronically injured BBB affect Aβ aggregation in turn? From this, the mechanisms of Aβ formation, transportation, and clearance, as well as the functions of a series of Aβ transporters on BBB were summarized, thus providing several potential targets for future investigation.

The fourth review focused on the hot issue of AD treatment (Hu and Wang). The authors first provided an up-to-date review on the different theories proposed for the pathogenesis of AD, especially on neuroinflammation, in which microglia, astrocytes, NK cells, and T cells are involved. Then, they summarized the functions and mechanisms of stem cell therapy, which is the only treatment approach so far that has pleiotropic therapeutic effects for multiple mechanisms in AD. In addition, mesenchymal stem cells are currently the most widely used stem cell type in AD clinical trials. The authors also explored the ongoing major mesenchymal stem cell clinical trials and showed how translational stem cell therapy is bridging the gap between basic science and clinical intervention in neurodegenerative diseases. Considering no drug is currently available to cure the disease or slow down its progression, this paper is strongly recommended for life science scholars in the field of stem cell research, and for translational medicine researchers who are interested in AD.

While the four reviews have enriched our previous understanding of the exact role of chronic inflammatory response in the development of neurodegeneration under various pathological conditions, the upstream regulation mechanisms still remain to be elucidated. Further studies from *in vitro* to *in vivo* models need to be conducted using new techniques, such as single cell sequencing, spatial transcriptomics, and multi-omics analysis, with the hope of identifying potential therapeutic targets for clinical application.

## Author Contributions

XG wrote the manuscript, acted as an editor to this Research Topic, and selected the articles described herein. All editors agreed to authorize XG to write this editorial on behalf of the guest editorial team, and approved the submitted version.

## Funding

This research was supported by grants from the National Natural Science Foundation of China (No. 82071394) and the Science and Technology Project of Tianjin Municipal Health Commission (No. TJWJ2021QN005).

## Conflict of Interest

The author declares that the research was conducted in the absence of any commercial or financial relationships that could be construed as a potential conflict of interest.

## Publisher's Note

All claims expressed in this article are solely those of the authors and do not necessarily represent those of their affiliated organizations, or those of the publisher, the editors and the reviewers. Any product that may be evaluated in this article, or claim that may be made by its manufacturer, is not guaranteed or endorsed by the publisher.
